# Cancer and orofacial pain

**DOI:** 10.4317/medoral.21515

**Published:** 2016-10-01

**Authors:** Marcela Romero-Reyes, Daniela Salvemini

**Affiliations:** 1 DDS, PhD. Assistant Professor. Orofacial and Head Pain Service, Department of Oral and Maxillofacial Pathology, Radiology and Medicine, New York University College of Dentistry, New York; 2 PhD, Professor. Department of Pharmacology and Physiology, Saint Louis University School of Medicine, 1402 South Grand Blvd, South Grand Blvd, St. Louis, USA

## Abstract

**Background:**

Cancer pain is a devastating condition. Pain in the orofacial region, may be present as the single symptom of cancer or as a symptom of cancer in its later stages. This manuscript revises in a comprehensive manner the content of the conference entitled “Orofacial Pain and Cancer” (Dolor Orofacial y Cancer) given at the VI Simposio International “Advances in Oral Cancer” on the 22 July, 2016 in Donostia.

**Material and Methods:**

We have reviewed (pubmed-medline) from the most relevant literature including reviews, systematic reviews and clinical cases, the significant and evidence-based mechanisms and mediators of cancer-associated facial pain, the diverse types of cancers that can be present in the craniofacial region locally or from distant sites that can refer to the orofacial region, cancer therapy that may induce pain in the orofacial region as well as discussed some of the new advancements in cancer pain therapy.

**Results:**

There is still a lack of understanding of cancer pain pathophysiology since depends of the intrinsic heterogeneity, type and anatomic location that the cancer may present, making more challenging the creation of better therapeutic options. Orofacial pain can arise from regional or distant tumor effects or as a consequence of cancer therapy.

**Conclusions:**

The clinician needs to be aware that the pain may present the characteristics of any other orofacial pain disorder so a careful differential diagnosis needs to be given. Cancer pain diagnosis is made by exclusion and only can be reached after a thorough medical history, and all the common etiologies have been carefully investigated and ruled out. The current management tools are not optimal but there is hope for new, safer and effective therapies coming in the next years.

**Key words:**Pain, orofacial, facial, cancer.

## Introduction

Pain due to cancer is a devastating consequence for many patients worldwide. Pain is defined as an unpleasant sensory and emotional experience associated with actual or potential tissue damage, or described in terms of such a damage as described by the International Association for the Study of Pain. Pain, IASP Pain Terminology, 1994. The pain experience is fundamentally protective and this quality can be illustrated when it is the first symptom of cancer. However, the pain experience can become maladaptive in response to damage or in the absence of an imminent damage, and as a result, not achieving its role as a protective mechanism.

Orofacial pain can arise as a symptom of regional or distant cancers resulting from nociceptive/somatic, neuropathic, inflammatory and visceral mechanisms (1-3). It is important to underscore that the characteristics of the pain can imitate the symptomatology of any orofacial pain disorder with descriptors of dull, aching, sharp, shooting, stabbing, burning, pulling, and throbbing pain, and refer pain to any craniofacial structure regionally or from a distant site (3). Therefore, the clinician needs to be aware of the diverse orofacial pain disorders and to understand that cancer pain must be included in the differential diagnosis of patients with unexplained and intractable orofacial pain.

## Key players in oral cancer pain

The pathophysiology of cancer pain is very complex. Unfortunately, there is still a lack of understanding of the mechanisms of cancer pain that has prevented the advancement of new targeted therapies due to the different etiologies of the diverse types of cancers, the tumor microenvironment and the anatomic location of the cancer per se (4). Pain may be the consequence of a primary, systemic or metastatic cancer and may reflect changes in both peripheral and central nervous systems.

Different mechanisms are involved in the pathophysiology of cancer pain (3,4). Cancer cells have the ability to infiltrate nerves along the epineural, perineural and endoneural space, inducing tissue infiltration and nerve damage, and this is known as perineural invasion (PNI) (5,6) and is associated with tumor progression and local recurrence (6,7). Not all cancers lead to PNI, but it is reported in up to 80% of head and neck cancer patients, the most affected nerves are the trigeminal and the facial nerve (8). PNI is present in squamous cell carcinoma, adenoid cystic carcinoma, lymphoma, and rhabdomyosarcoma (8,9).

Oral cancers are able to secrete mediators in the surrounding microenvironment in an autocrine and paracrine manner supporting cancer proliferation and metastasis (10,11). These mediators have a nociceptive quality and are able to directly excite and sensitize primary afferent neurons innervating the cancer microenvironment (12). Cancer-derived nociceptive mediators include inflammatory cytokines such as TNF-ɑ and other interleukins, neurotrophic factors, ATP, protons, proteases, and endothelins (12-14).

Approximately 90% of the head and neck cancers are squamous cell carcinomas (SCC) (15). Oral squamous cell carcinoma (OSCC) is commonly localized in the tongue (16), is severely painful and spontaneous facial pain has been reported as a primary symptom (15-17). Endothelin-1 (ET-1) has nociceptive effects in the tumor microenvironment and it is highly secreted in OSCC and targeting ET-1 in pre-clinical models has shown to have anti-nociceptive effects (18-20). In addition, OSCC also produces nerve growth factor (NGF) which has nociceptive properties. In pre-clinical models, targeting NGF has been shown to relieve cancer pain, cachexia and progression (14). High levels of ATP have been shown in human head and neck SCC (HNSCC) tissue. The tumor microenvironment of HNSCC is heavily innervated by nerve fibers expressing both P2X2 and P2X3 receptors that may have target potential, since ATP causes their activation and NGF induces their hypersensitivity at the level of the trigeminal ganglion (13). Chronic cancer pain is associated with elevated serine proteases in the tumor microenvironment and the protease-activated receptor 2 (PAR2) present in peripheral nerves has been shown to play an important role. In a transgenic model of PAR2-deficient mice, the development of chronic cancer pain was prevented (21).

## Orofacial pain as a symptom of cancer

It is very important to recognize that cancer in the orofacial region not only presents spontaneous pain but pain with function affecting significantly the quality of life of the patient. The normal biomechanics of eating, drinking, swallowing and talking could be compromised and be painful.

- Head and neck cancers

The onset of orofacial pain that is exacerbated during normal oral function is a key predictor for the transition from oral precancer to cancer (22). Regional orofacial pain and other sensory disturbances occur in 80% of patients with head and neck cancers (23). It is important to note that perineural spread of head and neck tumors can give symptomatology of trigeminal neuropathic pain, with sensation of burning, tingling, achy feelings as well as neuralgic symptoms such as sharp shooting electrical pains, and be triggered by function and even to present symptomatology of neurovascular disorders such as headache (9,24). A critical step is that a careful examination should be considered since the pain can be reported in any structure of the craniofacial region such as a toothache, pain in the gingiva, tongue, face, neck, ear and palate (17). The pain can be referred to the temporomandibular joint (TMJ) and be described as dull aching pain and present all the signs and symptoms of temporomandibular disorders (TMD), and therefore it could be misdiagnosed (25,26). Furthermore, intracranial tumors may present headache and orofacial pain symptoms such as trigeminal or glossopharyngeal neuralgia, so neuroimaging must be considered to confirm the diagnosis (27). It is important to recognize that symptoms of TMD, trigeminal neuralgia and persistent idiopathic facial pain are the three most common pain presentations in patients with intracranial tumors that come to the dental office (28).

- Metastasis

It has been reported in a retrospective study that of 114 cases of metastatic tumors in the jaws, in 60% of these cases the lesion was the only indication of a primary malignancy elsewhere (29). Malignancies originating from thyroid, esophagus, breast, lung, kidney, liver, female reproductive system, prostate, colon and rectum can metastasize to the orofacial region (30-32). Bone metastases such as in the mandible present persistent pain, swelling and other sensory disturbances (30,33). It is very important to consider that symptomatology resembling trigeminal neuralgia has been reported as a symptom of prostate cancer when the metastatic lesion involved the mandible (34), and in breast cancer when it involved the pterygopalatine fossa (35). Lung and breast malignancies can metastasize to the TMJ, and TMJ pain may be the first symptom of metastasis (36).

- Non-metastatic tumors

Orofacial pain referred from non-metastatic cancer is very rare but it has been reported that lung cancer and mediastinal malignant disease secondary to lung cancer can refer orofacial pain in the ipsilateral side (37,38). This referred pain could be provoked by compression of the vagus nerve by the lung (or any other organ or structure along the nerve), causing a convergence of somatic and visceral afferent inputs to the trigeminal nucleus caudalis, causing pain symptomatology in these regions (39). The pain has been described as intractable, unexplained, debilitating, severe, aching and paroxysmal, and with a poor response to therapy (37,38,40,41). Furthermore, neuralgic symptoms such as trigeminal neuralgia have been reported as the only symptom of pancreatic cancer (42).

- Systemic Cancer

Lymphoma, leukemia and myeloma are common neoplasias and they can be painful when they infiltrate bone, gingiva and when in close proximity to teeth (43-46). The osteolytic lesions present in multiple myeloma can induce odontogenic and bone pain, swelling of the area, root resorption, and tooth mobility, therefore, careful consideration needs to be given to rule out an odontogenic cause or systemic disease (46). These systemic cancers, in addition to pain, are associated with other neurosensory symptoms, such as the numb chin syndrome (NCS), which is a neuropathy described by numbness and hypoesthesia in the mental nerve distribution (47) and can present neuroleukemiosis (48,49).

- Paraneoplastic Syndrome

This phenomena can be present in response to breast cancer, gynecologic tumors, small cell lung cancer and hematologic malignancies (50). Paraneoplastic neuropathies or paraneoplastic syndrome refers to signs and symptoms similar to an autoimmune response that attacks the nervous system as a result of the presence of cancer, but not as a result of the local mass (3). These types of neuropathies are rare in the orofacial region (51), but trigeminal pain with a history of diarrhea and astenia has been reported in response to small cell lung carcinoma (52).

## The paradox of cancer treatment

New scientific paradigms and protocols are being implemented for cancer therapies that have extended the life of the cancer patient with new advancements in chemotherapy and radiotherapy. However, these immunotherapeutic approaches to restore the survival and function of the immune effectors, in addition to tumor specific targets to eradicate cancer without causing damage to healthy organs and tissue, remain a challenge (53-55). Therefore, the paradox rests in that extending the life of the cancer patient may extend the duration of experiencing pain and therefore, living with a quality of life less than optimal.

Pain as a consequence of cancer therapy is a very unfortunate problem. In a systematic review from 52 studies, 59% of cancer patients presented pain with anticancer treatment, 33% after cancer treatment, and more than one third of cancer patients reported their pain as moderate to severe (56). Chemotherapy can produce severe peripheral neurotoxicity leading to neuropathic pain (57). Orofacial pain of neuropathic origin can arise as a consequence of surgery (tumor resection), chemotherapy, and radiotherapy, or combination therapy (1,58). Most of the patients undergoing chemotherapy and radiotherapy for head and neck cancers develop oral mucositis which is extremely painful (59,60). The quality of life for these patients gets severely diminished since for some of them it is too painful to eat, so an adequate nutrition is compromised. In addition, xerostomia can be present making them more susceptible to rampant caries and also they are more susceptible to candidiasis and herpetic infections (59,61,62).

The current management of orofacial pain in cancer pain patients is similar to cancer pain in other parts of the body (51). Management involves anticonvulsants, antidepressants, NMDA antagonists, opioids, cannabinoids, topical agents and local anesthetics (63). For oral mucositis, management also includes mouthwashes with antimicrobial, analgesic, anesthetic and anti-inflammatory properties, as well as oral mucosal protectants, to create a protective shield against irritation (59,61). The WHO analgesic ladder has shown these approaches to be successful in achieving adequate pain control in 80-90% of patients, but there is not enough data about the control of orofacial pain independent of oral mucositis (non-mucositis pain) (64). Medications such as opioid regimens may reduce the pain for some patients but the development of tolerance and side effects are challenging for patients when larger dosages need to be administered. Therefore, new treatment approaches that manage the pain without sacrificing the cancer targeted therapy are urgently needed.

Moreover, musculoskeletal complaints can arise after head and neck surgery and radiation. Myofascial pain can be present as trismus, contracture, fibrosis and scarring of the muscles of mastication and TMJ ligaments (61,65-67) compromising daily life activities such as eating because they can cause severe limitations of mouth opening. Therefore, it is recommended that the patient begins a physical therapy protocol before and after the procedure to maintain an optimal mouth opening.

## New treatments, new hopes

Chemotherapy induced peripheral neuropathy (CIPN) and chronic neuropathic pains including burning oral dysesthesia as well as oral mucositis, are major side effects that warrant dose reduction of the antitumor agent and therefore affecting greatly the cancer prognosis (68,69). An ideal anti-tumor treatment should offer no painful side effects without sacrificing effective anti-tumor effects overall stabilizing the quality of life of the patient in their pathway to health. New understanding in CIPN may provide the foundation for effective treatments and offer new hopes in the battle against cancer pain.

Paclitaxel is an effective and widely used chemotherapeutic indicated for treating non-small cell lung carcinomas, Kaposi’s sarcoma, breast and ovarian cancer and head and neck cancer (70,71).

Peroxynitrite (PN) is a powerful pro-nociceptive and nitroxidative species that has been shown in the induction and the maintenance of persistent pathophysiological pain (72,73). It has been reported that PN production in response to activation of nitric oxide synthases and NADPH oxidase in the spinal cord contributes to the neuropathological changes involved in paclitaxel induced CIPN. In a preclinical model of CIPN induced by paclitaxel, was demonstrated that targeting PN can not only reverse but prevent the formation of CIPN without jeopardizing the anti-tumor effects of paclitaxel (71). Therefore the development of PN-targeted therapeutics may offer a new avenue for 

management. Moreover, pre-clinical studies have shown that A3 adenosine receptor (A3AR) agonists have antinociceptive effects (74-77). It has been demonstrated that activation of the A3AR with selective A3AR agonists blocked the development of CINP induced by different chemotherapeutics, without interfering with anticancer effects by inhibiting key pathways known to drive central sensitization (74,76-79). Treatment with C1-IB-MECA an A3A agonist reduced tumor growth as well as bone related pain as shown in rodent models of breast cancer bone metastasis (77,80). Moreover, MRS55698 a newer generation and highly selective A3AR agonist has shown to be effective in chronic pain states (81). In regards to human studies, it has been shown that in trials for inflammatory conditions including glaucoma, hepatitis, psoriasis and rheumatoid arthritis, A3A agonists offer tolerability and a good therapeutic index supporting an exciting avenue for their use in the management of chronic pain (82). Recently, a I/II clinical trial by Can-Fite BioPharma showed that C1-IB-MECA is successful as an anti-tumorigenic against hepatocecellular carcinoma (75), so the use of these agonists may offer a dual effect treating the cancer as well as the pain in cancer.

A great promise in regards this new target is that the effect of these A3AR agonists is independent of endocannabinoids or opioid pathways to exert their anti-nociceptive function as shown in pre-clinical models (77). A3AR are not subjected to analgesic tolerance and do not create reward (77). Therefore, the use of A3AR agonists may be a novel avenue that will offer to the cancer patient a safer, more tolerable, anti-cancer drug with anti-nociceptive properties without the risk of tolerance or abuse.

## Conclusions

Cancer pain is a devastating consequence of the cancer itself and unfortunately a sequela of cancer treatment. Cancer pain patients need a compassionate, multidisciplinary team for their management. The quality of life in head and neck cancer patients decreases dramatically since because of the pain they present challenges in ea-ting, talking swallowing and breathing and sometimes because of the same pain a dose reduction of the cancer therapy is needed, affecting in this way their cancer prognosis of survival. Preventive modalities should be implemented when orofacial pain is present as a result of cancer therapy therefore, in the cases of radiation or surgical procedure, a physical therapy protocol before and after the procedures is imperative to maintain a functional mouth opening. There is still a lot to understand in regards cancer pain but new advancements in potential targets are on the horizon that promise to be potentially beneficial. As clinicians we need to be aware that tumor size generally is not relevant in correlation with pain severity. In addition, cancer related pain in the orofacial region can be a symptom of a local tumor or a distant tumor and can present the same characteristics of the different orofacial pain disorders, mimicking these disorders, such as TMD or trigeminal neuralgia or neuropathic pain. Since sometimes pain can be the single symptom of cancer and in some cases it can be the first symptom of cancer, or a symptom of later stages, it needs to be underscored that pain itself should not be used as the only diagnostic criteria for cancer. Cancer pain diagnosis is made by exclusion and only can be reached after a thorough medical history, and all the common etiologies have been carefully investigated and ruled out (3). When in doubt, always seek the referral to an orofacial pain specialist for further evaluation.
